# Tree cover increases on agricultural land amid continued landscape-level deforestation: a case study from eastern Afghanistan

**DOI:** 10.1007/s10661-026-16018-1

**Published:** 2026-10-16

**Authors:** Farzaneh Razmju, Baoling Gui, Lydia Sam, Anshuman Bhardwaj, Mohammad Waris Haider, Safiullah Khurram, John W. Groninger, David F. R. P. Burslem

**Affiliations:** 1 School of Biological Sciences, University of Aberdeen, Aberdeen, UK; 2 Agriculture Faculty, Woman Online University, Northwood, UK; 3 The Hong Kong Polytechnic University, Hung Hom, Kowloon, Hong Kong SAR China; 4 School of Geosciences, University of Aberdeen, Aberdeen, UK; 5 Indian Agricultural Research Institute, New Delhi, India; 6 Department of Forestry and Natural Resources, Faculty of Agriculture, Kabul University, Kabul, Afghanistan; 7 School of Forestry and Horticulture, Southern Illinois University, Carbondale, IL USA

**Keywords:** Land use and land cover change, Tree cover, Agroforestry, Object-based image analysis, Sankey diagrams, Afghanistan

## Abstract

Understanding changes in tree cover within agricultural landscapes is critical for assessing the resilience of rural communities and consequent land use dynamics in conflict-affected regions. This study examines spatiotemporal land use and tree cover changes across four districts of Nangarhar Province in eastern Afghanistan using high-resolution (3 m/pixel) satellite imagery. The temporal window examined for this study (2016–2024) spans a period of significant geopolitical upheaval in Afghanistan, including the collapse of the republican government in 2021 and the resulting shift in political power and governance dynamics. An object-based image analysis framework combined with a support vector machine classifier was applied to map five land use classes within settlement areas: tree cover, cropland, water, built-up areas, and barren land. Classification accuracy was assessed using stratified random sampling and Cohen’s kappa coefficient, with all maps achieving *κ* values above 0.80. Post-classification change detection and transition matrices were used to quantify class-to-class conversions in hectares, while Sankey diagrams were employed to visualise the magnitude and direction of land use transitions. The results reveal varying patterns of tree cover change across the study districts, with evidence of both tree cover gains linked to expansion of agroforestry and on-farm tree retention, and losses driven by agricultural expansion and development. These changes may influence local livelihoods, as trees on farms provide important resources such as fruit, fuelwood, and shade. The findings demonstrate the effectiveness of integrating high-resolution remote sensing, object-based classification, and Sankey-based visualisation for analysing complex land use dynamics.

**Supplementary information:**

The online version contains supplementary material available at https://doi.org/10.1007/s10661-026-16018-1.

## Introduction

Globally, pressures on natural resources are often intensified in regions affected by conflict, where governance structures weaken, poverty deepens, and communities become increasingly dependent on nearby forests for survival (Daiyoub et al., 2023). Afghanistan represents one such context. Decades of conflict and political instability have undermined natural resource management systems, enabling widespread extraction of timber and fuelwood and accelerating forest degradation (Khurram et al., 2024; Shalizi et al., 2018). In the eastern provinces of Kunar, Nuristan, and Nangarhar, substantial forest loss occurred between 1977 and 2002, while illegal logging, overgrazing, agricultural encroachment, and poor regeneration further contributed to long-term deforestation and landscape degradation (Bader et al., 2013; Khurram et al., 2024; United Nations Environment Programme [UNEP], 2003). For many rural households, fuelwood scarcity remains a persistent challenge, and reforestation and afforestation within relatively secure agricultural landscapes are increasingly viewed as important strategies for restoring vegetation, stabilising soils, and reducing pressure on remaining forests (Groninger, 2012; Khurram et al., 2026; Milbrandt & Overend, 2011). Taken together, these conditions suggest that, in such conflict-affected environments, agricultural land may provide more secure and accessible spaces for tree planting and long-term vegetation management than remote or weakly governed forest landscapes.

Trees on farms provide well-documented benefits for rural livelihoods and agroecosystem functioning, including food, fuelwood, fodder, timber, carbon sequestration, soil protection, and microclimate regulation (Dogbo et al., 2024; Kuyah et al., 2016; Legesse & Negash, 2021). The number, diversity, and distribution of trees on farms are shaped by a combination of socioeconomic, ecological, and management factors (Endale et al., 2017; Legesse & Negash, 2021). However, the establishment and adoption of trees on agricultural land are often constrained by insecure land tenure, limited access to planting material, small landholdings, and inadequate extension support (Bosshard et al., 2026; Mukoobwa et al., 2023). Conversely, improved market access, secure tenure, and targeted support programmes can promote tree planting and increase on-farm tree diversity (Derero et al., 2021; Do et al., 2025; Gebreegziabher et al., 2020). Strengthening tree-based farming systems can therefore contribute to enhancing rural livelihoods, improving climate resilience, and supporting biodiversity conservation (Dogbo et al., 2024; Fuchs et al., 2022).

Afghanistan holds significant potential for agroforestry development. Although only about 13.2% of the country’s land is used as cropland, almost a quarter of the national territory has favourable conditions for integrating trees into farming systems (Ahmad et al., 2022). Many farmers, particularly in the south-east, rely on fruit and nut trees, shrubs supplying fodder, and other species well suited to local environments (Ahmadzai et al., 2024; Bader et al., 2013). Trees on farms have been widely shown to reduce soil erosion, enhance water retention and improve soil fertility, and contribute to waterway stabilisation and flood protection, thereby supporting higher crop yields and more resilient production systems (Cyamweshi et al., 2023; Judson et al., 2026; Tomer et al., 2015). By providing fuelwood, fodder, and other useful tree products directly on farmland, trees can also reduce pressure on natural forests. Studies from various regions highlight how on-farm trees offer resources that would otherwise be sourced from forests, thereby decreasing forest extraction (Kuyah et al., 2016; Rahman et al., 2017). Beyond farm-level benefits, agroforestry can enhance landscape connectivity and biodiversity, acting as a buffer around forest fragments (Sagastuy & Krause, 2019). Contributions to soil and biomass carbon further support climate change mitigation (De Stefano & Jacobson, 2018; Fuchs et al., 2022).

Recent evidence from Afghanistan indicates that farmers’ decisions to plant trees are influenced by socioeconomic and farm-level factors, particularly household income, agricultural landholding, access to irrigated land, and previous tree planting experience (Omerkhil et al., 2024). However, limited availability of seedlings, financial constraints, and insufficient extension services remain critical barriers. Strengthening the integration of trees into agroecosystems therefore requires targeted policies and improved support mechanisms. In Afghanistan, policies and programmes supporting tree-based farming and agroforestry have largely been implemented through government-led and donor-supported agricultural development initiatives (Khurram et al., 2024). Major programmes, including the National Horticulture and Livestock Project (NHLP), focused on orchard establishment, distribution of fruit tree saplings, farmer training, livestock support, and improvements to agricultural infrastructure, including irrigation systems (World Bank, 2021). Such interventions were widely implemented during the Republic period (2012–2021) and aimed to improve rural livelihoods while increasing agricultural productivity and resilience. Following the political transition in 2021, many development programmes experienced funding reductions, institutional restructuring, or changes in implementation arrangements (United Nations Development Programme [UNDP], 2022). As a result, support for tree-based farming and agroforestry activities became more limited and increasingly dependent on humanitarian and project-based interventions (Khurram et al., 2026). This temporal shift may have influenced the continuity and intensity of agroforestry-related support mechanisms across the study period.

Investments to support and expand agroforestry require monitoring, reporting, and verification (MRV) to understand the effectiveness of interventions over long timescales and to make adjustments to their design where needed (Rosenstock et al., 2019). However conventional field-based MRV approaches are challenging in conflict-affected regions where access and fieldwork are chronically difficult and dangerous (Daiyoub et al., 2023). In this paper, we test the efficacy of remote sensing technologies based on high-resolution satellite imagery to provide an effective means of assessing tree cover, tree density, and canopy condition over time, even in inaccessible areas, as a partial surrogate for standard approaches to MRV in agroforestry projects. Platforms such as PlanetScope, Sentinel-2, and Landsat enable continuous monitoring across large landscapes and offer reliable information on land use and land cover change (Hemati et al., 2021; Szostak, 2022). The integration of remote sensing with GIS further enhances landscape analysis and environmental monitoring (Laosuwan & Uttaruk, 2016; Rizvi et al., 2016).

Research on tree cover change within small-scale agricultural landscapes in conflict-affected contexts remains limited (Murillo-Sandoval et al., 2021; Vanegas-Cubillos et al., 2022). Remote sensing research in Afghanistan has often focused on broad-scale land cover and forest cover assessment, with comparatively less attention to trees within agricultural landscapes (Reddy & Saranya, 2017; Uddin et al., 2024). Evidence from Tigray, Ethiopia, demonstrates that conflict may result in rapid reductions in vegetation cover due to breakdowns in conservation practices and increased reliance on wood resources (Hishe et al., 2024), underscoring the importance of remote monitoring in insecure and difficult-to-access environments. In Afghanistan, remote sensing data have also played an important role in shaping land management and reforestation priorities, particularly where field access to critical landscapes has been constrained by insecurity and terrain (Ahmad et al., 2022; Groninger, 2012; Jacobs et al., 2015). However, comparatively little attention has been given to agricultural landscapes, despite their importance for livelihoods, local resource use, and their potential for more stable long-term vegetation management (Brandt & Stolle, 2021; Khurram et al., 2026). As such, a clear gap persists in understanding how tree cover on agricultural land responds to prolonged conflict, governance shifts, and socioeconomic change.

This study aims to address this gap by quantifying changes in tree cover on agricultural land between 2016 and 2024 in four selected districts of Nangarhar Province, eastern Afghanistan, a period marked by significant political transition. The selected districts were chosen because, collectively, they are broadly representative of the agricultural landscapes found across eastern Afghanistan. Together, they encompass a range of farming systems, including irrigated valley agriculture, rain-fed farming areas, and mixed cropland–orchard landscapes, allowing comparison of tree cover dynamics across contrasting environmental and land use settings. From 2017 to 2021, the former Afghan government promoted community-based forest management and tree planting programmes (Khurram et al., 2024; National Environmental Protection Agency [NEPA], 2019). The political changes following 2021 fundamentally altered governance and economic conditions (UNDP, 2022). The withdrawal of much international development assistance and changes in institutional structures affected rural livelihoods and may have influenced land use decisions and natural resource management practices (Khurram et al., 2024; UNDP, 2022). By analysing high-resolution satellite imagery, this research seeks to determine whether the legacy of the tree planting programmes promoted by the former Afghan government can now be observed in the dynamics of tree cover on farmland. The study also evaluates the suitability of remote sensing technologies for monitoring agricultural tree cover in regions where security concerns and limited accessibility make field-based monitoring difficult. We address the following specific questions.

i. Has tree cover on agricultural land in the selected districts of Nangarhar Province changed between 2016 and 2024?
ii. How effective are remote sensing and GIS-based approaches for monitoring changes in tree cover on agricultural land in regions where access for researchers is constrained?

## Materials and methods

### Study area description

The study sites are located in Nangarhar Province in eastern Afghanistan. Nangarhar has an average elevation of approximately 826 m above sea level and a subtropical climate encompassing both hot desert (BWh) and hot semi-arid (BSh) zones under the Köppen classification (Mohammadi et al., 2023). Although precipitation occurs predominantly during winter and spring, the climate also includes seasonal monsoonal rainfall during the summer months, contributing to interannual rainfall variability (Ahmad et al., 2025). These climatic conditions are important factors shaping regional land use patterns, agricultural productivity, and forest resource management. Over recent decades, the study area and similar mixed agricultural landscapes across eastern Afghanistan have experienced substantial land use and land cover (LULC) changes. These changes have occurred in the context of prolonged conflict, demographic change (including population growth, migration, and displacement), pressure on land and water resources, shifts in agricultural practices, changing governance structures, and fluctuations in development assistance and rural investment. Periods of insecurity have affected natural resource management, infrastructure maintenance, and access to agricultural support services, while political and economic transitions have influenced farmers’ livelihood strategies and land use decisions. As a result, agricultural expansion, changing vegetation cover, and increasing pressure on natural resources have become important features of landscape change across the region. Although substantial forest losses have been documented in the mountainous forest regions of eastern Afghanistan, primarily due to illegal logging, timber extraction, and conflict-related disturbances (Ingalls & Mansfield, 2017), the present study focuses on agricultural and settled landscapes where different land use dynamics may operate. Understanding vegetation change within these managed agricultural environments is particularly important because they support local livelihoods and may respond differently to political and economic change than natural forest systems. National-level assessments further document widespread reductions in forest, grassland, and water bodies, accompanied by the expansion of agricultural and built-up areas (Najmuddin et al., 2022).

**Fig. 1:**
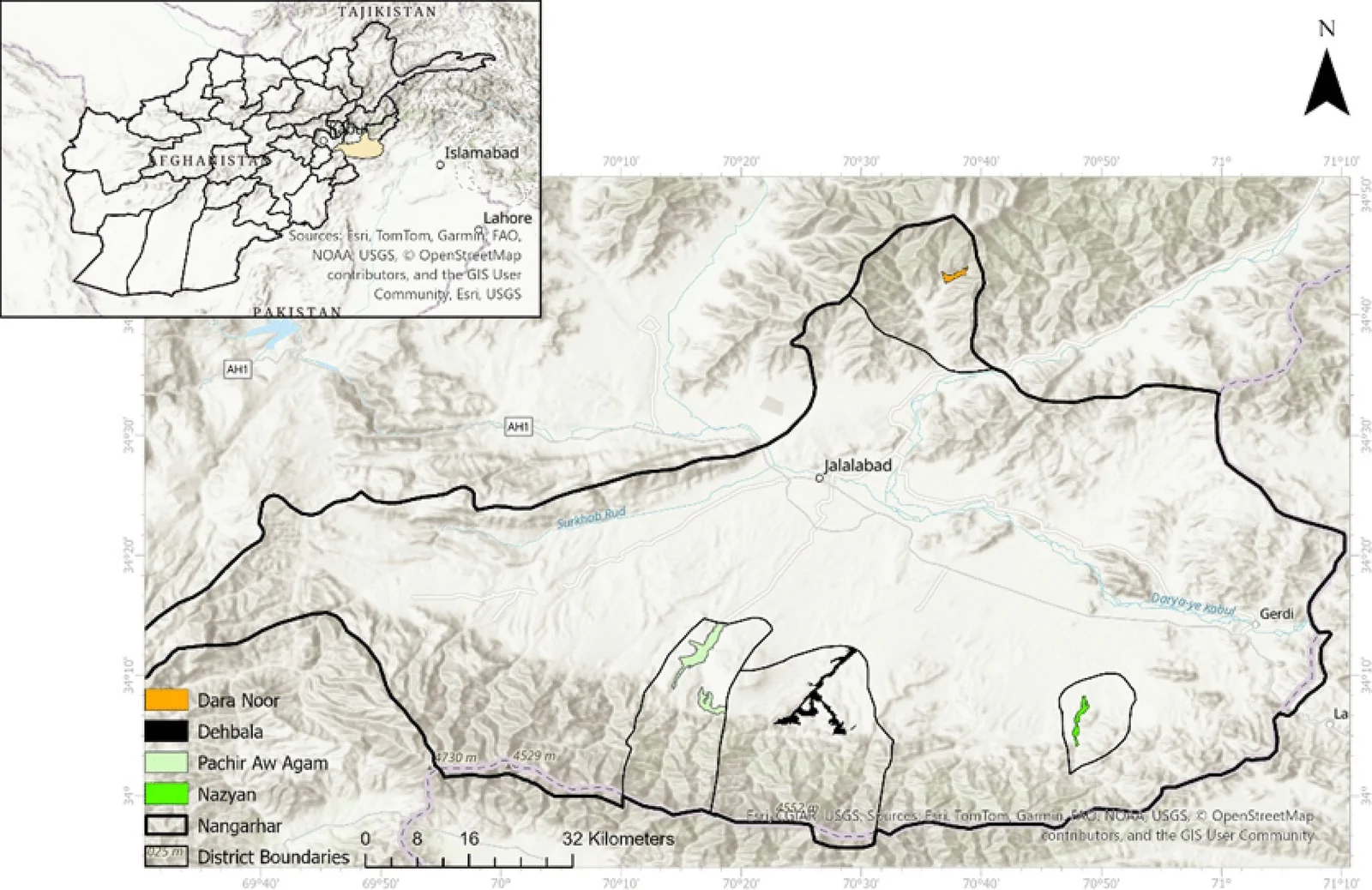
Study area: Nangarhar Province, eastern Afghanistan

**Fig. 2:**
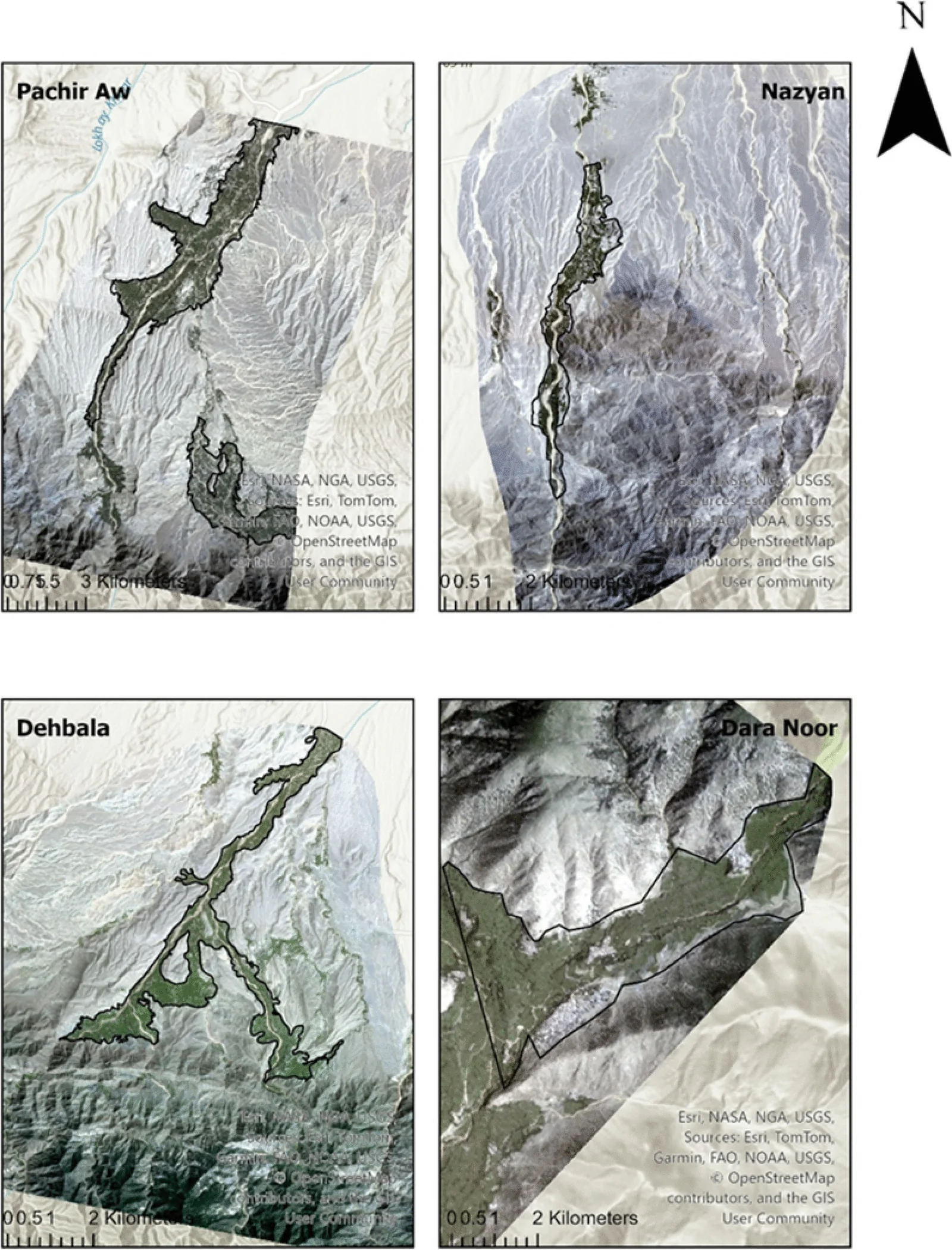
PlanetScope satellite images of study area across four districts, Nangarhar Province, Afghanistan

This study focuses on four districts within Nangarhar Province: Dara Noor, Nazyan, Dehbala, and Pachir Aw Agam (Fig. 1). These districts are predominantly mountainous but contain cultivated inter-montane valleys characterised by mosaics of cropland, orchards, scattered trees, settlements, and river corridors (Fig. 2). Collectively, they represent agricultural landscapes typical of eastern Afghanistan, where the seasonal influence of the South Asian summer monsoon contributes to regional climatic conditions (Ahmad et al., 2025).

The districts also differ in their elevation, local climatic conditions, and dominant land use characteristics (Fig. 2). Dara Noor contains mid-elevation humid valleys with remnant coniferous forests and mixed orchards, while Pachir Aw Agam represents a transitional zone between forested uplands and productive agricultural lowlands. In contrast, Nazyan and Dehbala are comparatively drier, with rain-fed agriculture, rangelands, and scattered orchards dominating the landscape.

Land use and tree cover changes were assessed using high-resolution PlanetScope imagery for the years 2016, 2020, and 2024 (Planet Labs PBC, 2026). A single fixed area of interest (AOI) was manually delineated for each district based on the agricultural areas within the valleys identifiable in the PlanetScope imagery. The AOIs encompassed cultivated fields and associated human-modified land, including tree patches, settlements, and river corridors. Tree patches within the AOIs consisted primarily of orchards and scattered farm trees, while areas dominated by rangeland, natural forest, and steep mountainous terrain were excluded because the study focused specifically on land use dynamics within agricultural and settled landscapes. The same AOI boundary was applied consistently to the 2016, 2020, and 2024 imagery for each district, ensuring that land cover changes were assessed over an identical spatial extent through time.

### Data acquisition and image pre-processing

PlanetScope satellite imagery for the years 2016, 2020, and 2024 was obtained from the Planet Explorer platform, with acquisition dates and image IDs provided in Table S1. Scenes were selected from the mid-to-late growing season (August–September) to minimise cloud cover and capture vegetation under peak canopy conditions. During this period, orchard crops, particularly grape and pomegranate, exhibit fully developed canopies, while the dominant agricultural land cover classes remain readily distinguishable. These conditions maximise spectral separability between cropland, tree cover, and other land cover classes, thereby improving classification performance. Each image has a spatial resolution of 3 m, making the dataset suitable for detailed land use and vegetation mapping at the farm level, including the identification of small tree patches, orchards, and field boundaries. Multiple image tiles for each year were mosaicked in ArcGIS Pro (Esri, n.d.) using the Mosaic to New Raster tool and reprojected to the WGS 84 UTM coordinate system. Area of interest (AOI) boundaries were digitised to define the spatial extent of analysis based on visually identifiable cultivated land. The mosaicked rasters were subsequently clipped to the AOIs to generate consistent spatial subsets for each district and study year.

### Training sample preparation

Training samples were generated using the Training Sample Manager in ArcGIS Pro. Five land cover classes were defined: tree cover, cropland, water, built-up areas, and barren land. Representative polygons were digitised for each class to capture within-class spectral and spatial variability. Tree cover included orchards, scattered farm trees, tree rows, and other areas where trees formed the dominant land cover feature. Built-up areas included roads, buildings, and other human-made structures, even where they occurred within orchards or agricultural land. Barren land comprised exposed soil, rock, and other areas lacking discernible vegetation cover. These samples were subsequently used for supervised classification and accuracy assessment.

To improve the reliability of training samples, visual inspection and validation were conducted using high-resolution historical imagery available in Google Earth Pro (Google LLC, n.d.). AOI boundaries were imported, and land cover types were cross-checked using imagery corresponding to the study years (2016, 2020, and 2024). This iterative verification process reduced classification uncertainty and improved the representativeness of training samples across heterogeneous agricultural landscapes.

### Image classification

Supervised classification was performed using the Supervised Classification Wizard in ArcGIS Pro. An object-based image analysis (OBIA) framework was adopted to minimise salt-and-pepper effects and to better capture spatial patterns in high-resolution imagery. Image segmentation was performed using the Segment Mean Shift algorithm in ArcGIS Pro. The same segmentation parameters were applied consistently across all four districts and three study years: spectral detail = 15.5, spatial detail = 15, and minimum segment size = 20 pixels. Active chromaticity colour and mean digital number were used as object attributes, while standard deviation and pixel count were additionally enabled to enhance object-level feature differentiation. Compactness and rectangularity were not used as object attributes.

The support vector machine (SVM) classifier was selected due to its demonstrated robustness in high-resolution remote sensing applications, particularly where training data are limited and landscapes are spatially heterogeneous (Huang et al., 2002; Khatami et al., 2016; Maxwell et al., 2018; Mountrakis et al., 2011; Pal & Mather, 2005). Given the sub-field spatial resolution of PlanetScope imagery and the limited availability of reference data, SVM was considered an appropriate classifier for this study. The SVM classifier was implemented using a nonlinear radial basis function (RBF) kernel. The C and gamma hyperparameters were automatically optimised within ArcGIS Pro using a two-dimensional grid search to identify the optimal parameter pair, rather than being manually specified (Esri,  n.d.). Consequently, the selected C and gamma values varied among the independently trained district-year classifications. The resulting hyperparameter values for each classification are provided in Table S2. Misclassified or uncertain regions identified during visual inspection were iteratively reviewed, and the training samples were refined where necessary to improve class separability.

### Accuracy assessment

Classification accuracy was evaluated using 500 stratified random validation points for each classified raster. Each validation point was compared against reference data derived from visual interpretation of high-resolution historical imagery available in Google Earth Pro (Google LLC, n.d.), following established good practices for accuracy assessment in remote sensing (Olofsson et al., 2014). Classification accuracy was evaluated using Cohen’s kappa coefficient (*κ*). Following established guidelines, *κ* values greater or higher than 0.80 were considered indicative of high classification reliability (Congalton & Green, 2009). Final LULC maps for 2016, 2020, and 2024 were symbolised using consistent colour schemes and exported as raster outputs for subsequent analysis.

### Land use change detection and transition analysis

Post-classification change detection was applied to assess land-use transitions between 2016, 2020, and 2024. Transition matrices were computed to quantify class-to-class conversions, with all transition areas calculated in hectares. These matrices provide a quantitative assessment of land use persistence and conversion across time periods.

To enhance the interpretability of land use dynamics, Sankey diagrams were employed to visualise both the magnitude and direction of land use transitions. In these diagrams, flow widths are proportional to the converted area, enabling an intuitive representation of complex class-to-class conversion pathways. This visualisation approach complements transition matrices by highlighting dominant land use change processes and facilitating a clearer understanding of temporal land use dynamics within the study area.

## Results

### Classification performance and accuracy assessment

Supervised classification using the SVM algorithm produced a clear separation among the five land cover categories defined as tree cover, cropland, built-up areas, water, and barren/other. Classification accuracy was assessed for each district using Cohen’s kappa coefficient (*κ*). As shown in Table 1, all districts achieved *κ* values above 0.80, indicating strong agreement with reference data. The highest kappa was recorded in Dehbala in 2020 (*κ* = 0.93), while the lowest was in Dara Noor in 2020 (*κ* = 0.80). These results confirm the reliability of the supervised classifications across all districts and study years.

**Table 1:** Cohen’s kappa coefficients (*κ*) for land cover classification in four districts in Nangarhar Province, Afghanistan, for 2016, 2020, and 2024. Following Congalton and Green, (2009), kappa values exceeding 0.80 are generally considered indicative of strong to almost perfect classification agreement

District	2016	2020	2024
Dehbala	0.84	0.93	0.88
Nazyan	0.89	0.89	0.88
Pachir Aw Agam	0.89	0.92	0.89
Dara Noor	0.94	0.80	0.87

### Tree cover change analysis

**Fig. 3:**
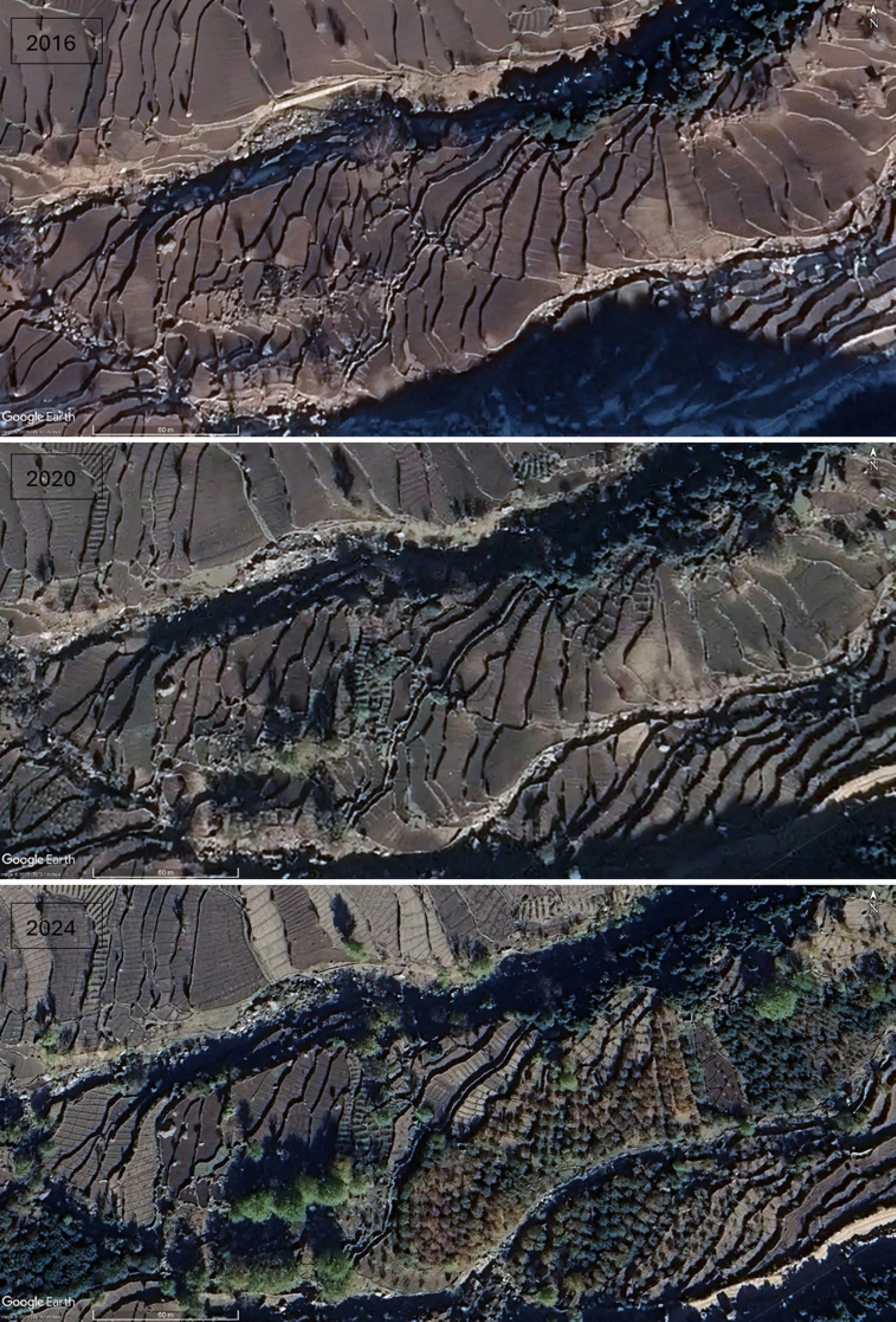
High-resolution imagery from Google Earth Pro showing changes in tree cover on agricultural land use in a subset of the Dara Noor District, Nangarhar Province, Afghanistan, in 2016, 2020, and 2024. All panels cover the same spatial extent to enable visual comparison of landscape changes

The spatial and temporal variations in tree cover across the study area were assessed using both absolute area (ha) and relative proportions (%) for the years 2016, 2020, and 2024 (Tables S3–S6). An example of localised changes in tree cover on agricultural land is illustrated in Fig. 3.

Across the four study districts, tree cover increased substantially over the study period. The total area classified as tree cover within the AOIs increased from 369.26 ha in 2016 to 502.58 ha in 2024, representing a net gain of 133.32 ha (36.1% of the 2016 value). Tree cover increased by 53.80 ha between 2016 and 2020 and by a further 79.52 ha between 2020 and 2024, indicating that the rate of expansion was greater during the latter period. This overall increase was observed across all four study districts, although the magnitude of change varied considerably among them (Tables S3–S6; Fig. 5).

In 2016, the total tree cover area within the AOIs was 369.26 ha (Table S3). Dehbala contained the largest extent of tree cover (216.96 ha), followed by Pachir Aw Agam (126.73 ha), while Dara Noor (12.75 ha) and Nazyan (12.82 ha) exhibited substantially smaller tree cover on agricultural land. When expressed as a proportion of the total area analysed, tree cover represented 10.21% of the AOI in Dehbala, 6.70% in Pachir Aw Agam, 4.61% in Dara Noor, and 3.10% in Nazyan (Table S5).

By 2020, the total tree-covered area within the AOIs had increased to 423.06 ha (Table S4). Absolute tree cover declined in Dehbala to 184.93 ha, while notable increases were observed in Pachir Aw Agam (190.65 ha) and Nazyan (34.36 ha). These changes increased tree cover proportions to 10.08% in Pachir Aw Agam and 8.32% in Nazyan, whereas tree cover decreased to 8.70% in Dehbala (Table S6). Dara Noor showed only a marginal increase in both absolute area and percentage tree cover during this period.

By 2024, tree cover had expanded further within the AOIs, reaching a total of 502.58 ha (Table S5). Dehbala recorded an increase in tree cover to 226.88 ha, corresponding to 10.68% of its total area. Nazyan exhibited the most pronounced relative increase, with tree cover reaching 17.03%, despite its smaller absolute area (70.34 ha). Pachir Aw Agam maintained a relatively high tree cover proportion (9.72%), while Dara Noor continued a gradual increase to 21.56 ha, representing 7.80% of its area (Table S6).

Although all districts showed higher tree cover on agricultural land in 2024 than in 2016, the magnitude and pattern of change differed among districts. Nazyan exhibited the largest relative increase in tree cover, particularly between 2020 and 2024, whereas Dehbala and Pachir Aw Agam displayed more moderate and fluctuating trends over the study period. Dara Noor showed a gradual but consistent increase with limited variability between the two study periods. Dehbala and Pachir Aw Agam consistently contained the largest areas of tree cover throughout the study period, whereas Nazyan and Dara Noor were characterised by comparatively lower baseline tree cover levels. Despite these differences, all districts exhibited the same general temporal pattern of increasing tree cover between 2016 and 2024 (Fig. 5).

### Land cover transition analysis

**Fig. 4:**
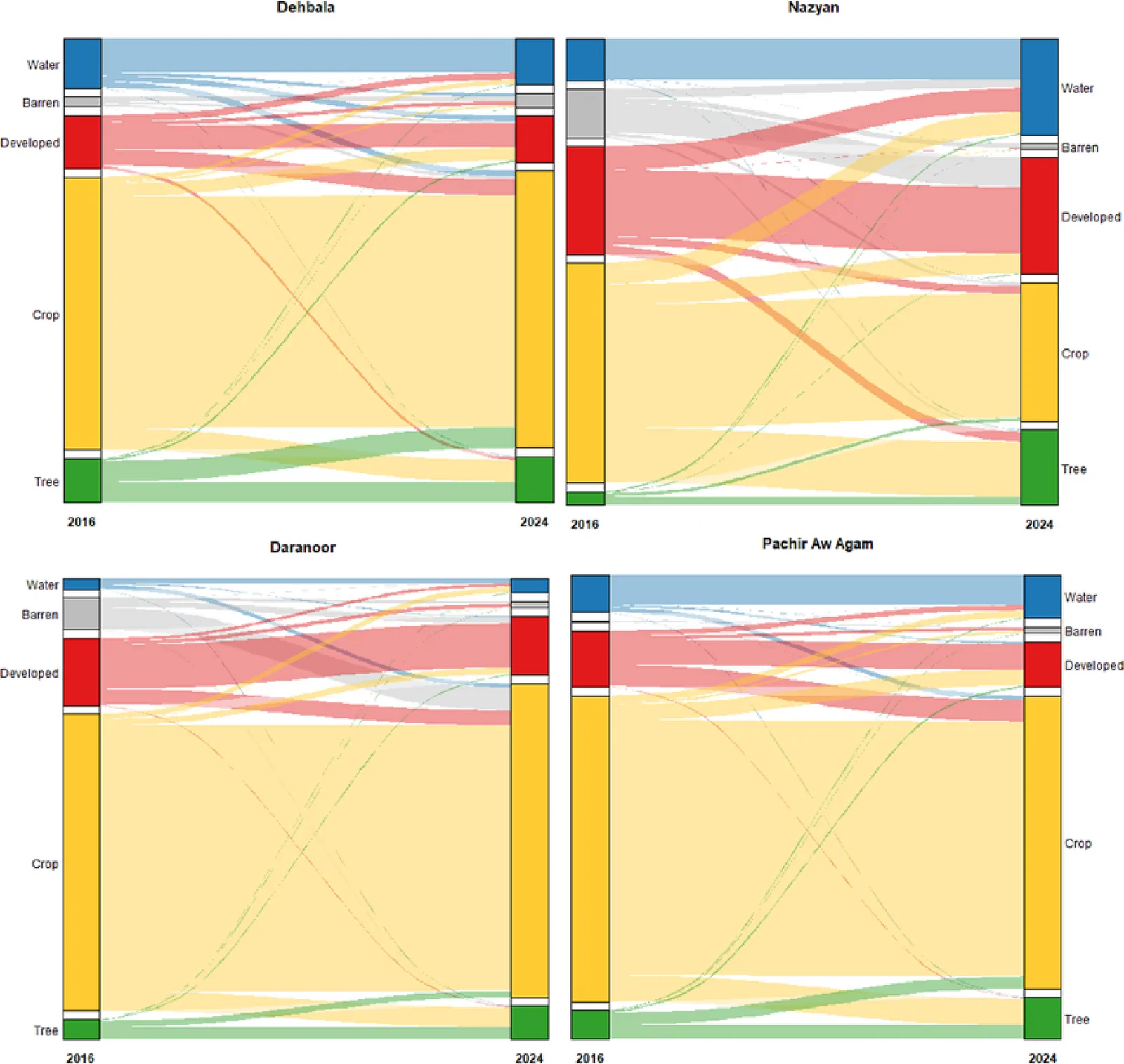
Sankey diagrams illustrating overall post-classification land cover transitions across the four districts between 2016 and 2024

Land cover transitions were analysed using post-classification comparison for two sub-periods (2016–2020 and 2020–2024) and for the overall study period (2016–2024) (Fig. 4 and Figs. S1–S2). Across all four districts, land cover dynamics were characterised by a pattern of overall structural stability combined with targeted transformations between classes.

Cropland was the most persistent land cover class throughout the study period, with the majority of agricultural areas retaining their classification. Built-up areas and water bodies also exhibited high stability, showing only limited exchanges with other land cover types. In contrast, the most significant dynamic process was the conversion of cropland to tree cover, which constituted the dominant pathway driving the observed expansion of tree cover areas. Smaller contributions to tree cover gain originated from barren land, while transitions from built-up areas were minimal. The smaller contribution of barren land to tree cover gain may similarly reflect either deliberate planting on previously unused land or natural woody regeneration where environmental conditions became more favourable. Distinguishing between these mechanisms is not possible using satellite imagery alone and would require field verification.

**Fig. 5:**
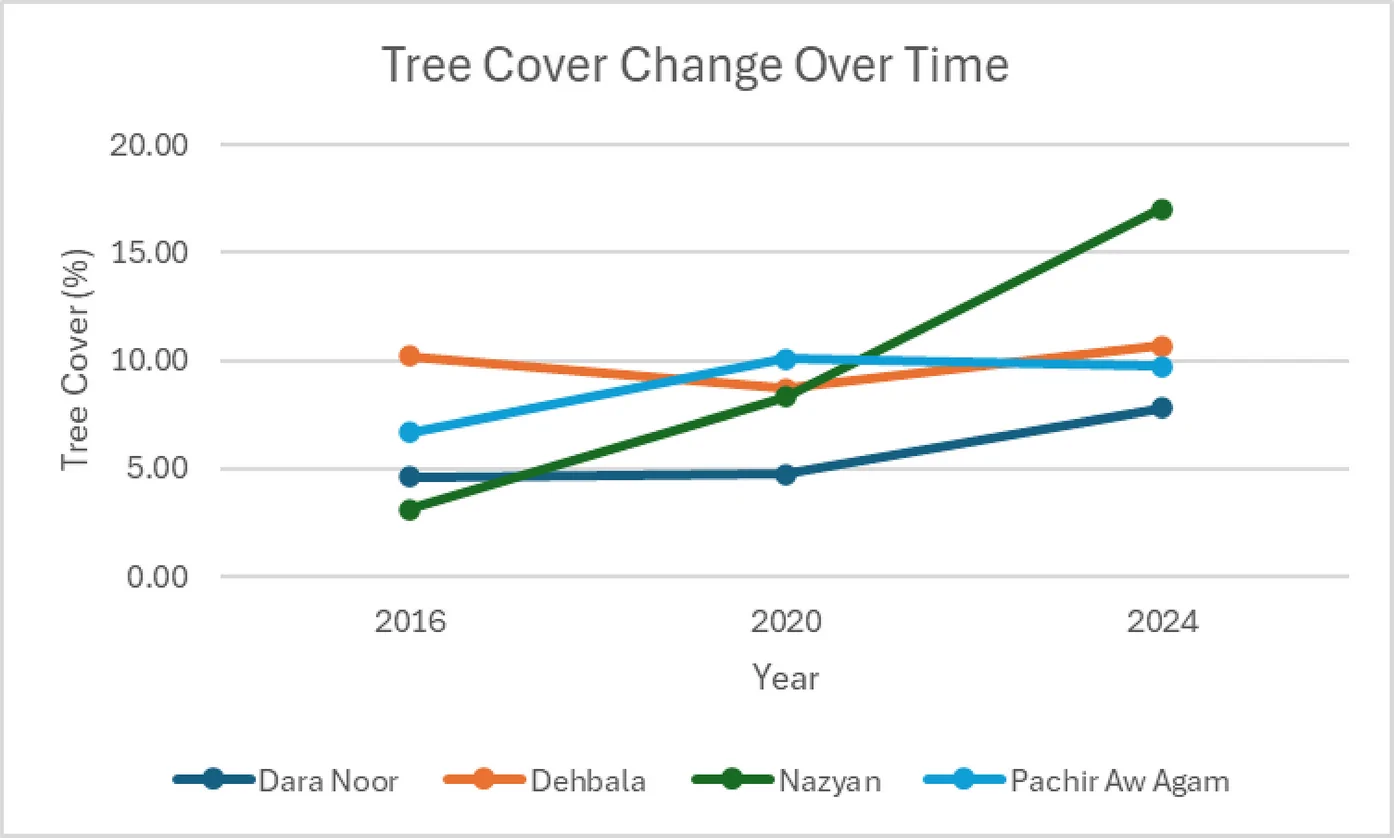
Tree cover change over time in four study areas

Comparison of the two sub-periods indicates that the overall structure of transitions remained consistent; however, the magnitude of cropland-to-tree conversion was more pronounced during 2020–2024. This suggests increased land cover dynamism in the later period, despite the persistence of core land use categories. Taken together, the results indicate that between 2016 and 2024, the study area experienced cumulative transformations of land cover primarily driven by agricultural land transitioning to tree cover, within a broader framework of land use stability (Fig. 5).

## Discussion

This study investigated changes in tree cover on agricultural land in a conflict-affected region of eastern Afghanistan between 2016 and 2024, with a particular focus on identifying both the magnitude and pathways of change. The results indicate a clear overall increase in tree cover across study sites within all four districts, with a net gain of approximately 133 ha (2.83% of the total study area) over the study period. Importantly, post-classification transition analysis reveals that this increase occurred primarily through direct conversions from cropland to tree cover, with smaller contributions from barren land, while conversions from built-up areas were minimal. These findings suggest that tree cover expansion has taken place largely within agricultural landscapes rather than through reductions in settlements or infrastructure. The observed increase is consistent with either deliberate tree planting by farmers, natural woody regeneration, or a combination of both processes, although the relative contribution of these mechanisms cannot be determined from the available data.

The increase in tree cover is notable given the broader national context of long-term forest degradation in Afghanistan. Previous studies and official reports indicate that Afghanistan has experienced severe deforestation over recent decades, particularly in the eastern provinces, owing to illegal logging, fuelwood extraction, and prolonged conflict (Ingalls & Mansfield, 2017; Milbrandt & Overend, 2011). Although Afghanistan has historically had relatively limited forest cover, historical assessments estimate that deciduous and evergreen forests covered approximately 5% of the country’s land area, with substantial forest areas concentrated in the eastern part of the country (Milbrandt & Overend, 2011). Eastern provinces such as Nangarhar, Kunar, and Nuristan have been particularly affected by illegal logging, fuelwood extraction, and conflict-related disturbances (Ingalls & Mansfield, 2017; Milbrandt & Overend, 2011). Against this background, the observed increase in tree cover on agricultural land between 2016 and 2024 represents a contrasting and locally significant trend.

Several factors may provide plausible explanations for this pattern, although their relative contributions cannot be determined from the remote sensing data. Between 2016 and 2021, the former Afghan government, together with international organisations, implemented a range of forestry, horticulture, and agroforestry-related projects aimed at restoring degraded land and improving rural livelihoods. Programmes such as the National Horticulture and Livestock Project (NHLP) expanded orchard development across multiple provinces, while FAO-supported initiatives promoted community-based forest management and agroforestry systems (Khurram et al., 2024; World Bank, 2021). Such interventions provide a possible contextual explanation for some of the observed tree cover expansion, particularly where programmes promoted fruit trees, poplar, and other multipurpose species (Gouhari et al., 2021; World Bank, 2021). However, the present analysis cannot establish whether, or to what extent, the observed increases resulted directly from these interventions.

An alternative possible explanation for the observed tree cover expansion is reduced cultivation intensity or temporary land abandonment, which could allow tree cover expansion through natural regeneration of woody plants. Such processes have been reported in conflict-affected settings, meaning that cropland-to-tree transitions do not necessarily indicate deliberate agroforestry adoption. Our study did not directly assess land abandonment or cultivation intensity. Distinguishing between active tree planting and passive regrowth would require field-based validation, which remains challenging in insecure environments (Daiyoub et al., 2023).

District-level differences further highlight the heterogeneous nature of land cover change. Nazyan exhibited the largest relative increase in tree cover, particularly during the later period (2020–2024), while Dara Noor showed more gradual but consistent increases. In contrast, Dehbala and Pachir Aw Agam displayed more moderate and fluctuating trends. These differences may be associated with variations in local socioeconomic conditions, access to irrigation, terrain, and farmers’ land use strategies, although these potential drivers were not directly assessed in this study. Previous research indicates that farm characteristics, household resource status, education, land tenure security, access to markets, and management-related factors can influence farmers’ decisions regarding tree planting and tree diversity in smallholder agricultural systems (Bosshard et al., 2026; Etongo et al., 2015; Legesse & Negash, 2021). These factors therefore provide plausible hypotheses for explaining the observed district-level variation, but their relative importance within the four study districts cannot be determined from the available data and would require direct socioeconomic investigation.

Nazyan presents a particularly striking example of land cover change within the study area. Between 2016 and 2024, tree cover increased from 3.10% to 17.03% of the analysed area, while the area classified as water increased from 9.75% to 21.29% (40.26 to 87.96 ha). Over the same period, cropland declined substantially from 50.88% to 32.77%. These concurrent changes indicate substantial reorganisation of land cover within the Nazyan AOI, although the present analysis does not provide hydrological, precipitation, or irrigation data with which to determine whether the increase in the mapped water class was related to the observed expansion of tree cover. Visual inspection of the high-resolution imagery indicates the presence of both scattered trees within or along the boundaries of agricultural fields and larger tree cover patches, some of which appear consistent with orchard systems. However, the available imagery does not allow reliable discrimination between newly established orchards and natural woody regeneration. Both deliberate tree planting and passive vegetation recovery remain plausible explanations for the observed increase in tree cover, but their relative contributions cannot be determined from the available data. Field observations, together with hydrological and irrigation data, would be required to evaluate the mechanisms underlying the distinctive land cover changes observed in Nazyan.

The findings are also consistent with broader evidence on the potential role of agroforestry in smallholder agricultural systems. Trees integrated into agricultural land can provide multiple benefits, including food, fuelwood, fodder, and income, while also contributing to soil fertility, moisture retention, microclimate regulation, biodiversity conservation, and the adaptive capacity of farming systems (Cyamweshi et al., 2023; Fuchs et al., 2022; Kuyah et al., 2016). Evidence from southeastern Afghanistan further shows that agroforestry systems provide a range of forest and tree products, including fruits, timber, wood, and pine nuts, and that farmers’ agroforestry practices are associated with socioeconomic conditions and perceptions of land use change (Ahmadzai et al., 2024). Agroforestry may also help reduce pressure on natural forests by providing alternative sources of tree products within agricultural landscapes; for example, farmers practising agroforestry in Indonesia were found to be less involved in forest clearing and forest product collection than swidden farmers (Rahman et al., 2017). In the context of continued degradation of Afghanistan’s natural forests, one possible hypothesis is therefore that the expansion of trees on agricultural land may partly reflect greater reliance on farm-based tree resources. However, any relationship between declining availability of forest-derived products and increasing tree cover on farms remains a hypothesis that would require household-level and field-based investigation.

Political and institutional changes after 2021 may also have influenced land use dynamics during the later study period. Following the political transition in August 2021, Afghanistan experienced substantial socioeconomic disruption and changes in livelihoods and economic conditions (UNDP, 2022). The reduction in international development projects and formal extension services following the change in government may have constrained new tree planting initiatives. At the same time, increased reliance on local resources may have reinforced the importance of trees on farms as sources of food, fuel, and income. Population movements associated with political and economic instability may also have altered rural livelihood and land use strategies, although their extent within the four study districts remains unknown. Evidence from other conflict-affected regions demonstrates that armed conflict can substantially alter vegetation and land use dynamics; for example, conflict in Tigray, northern Ethiopia, was associated with vegetation degradation and disruption of landscape restoration processes (Hishe et al., 2024). Thus, the post-2021 political transition represents a potentially important context for interpreting the observed changes in Nangarhar, but its contribution to the increase in agricultural tree cover remains to be determined by future research.

From a methodological perspective, this study demonstrates the value of remote sensing and GIS for monitoring tree cover dynamics in insecure and data-scarce contexts. Ground-based surveys, while detailed, are often costly, time-consuming, and impractical in conflict zones or mountainous terrain (Aziz et al., 2024; Hishe et al., 2024). In contrast, satellite imagery from platforms such as PlanetScope, Landsat, and Sentinel-2 provides consistent, repeatable, and objective data for tracking land cover change over time (Hemati et al., 2021; Szostak, 2022). Remote sensing studies in Afghanistan have often focused on natural forests, the present analysis highlights the feasibility and importance of extending such approaches to trees on agricultural land. Monitoring trees outside forests is essential for understanding landscape-scale vegetation dynamics and the role of agroforestry in post-conflict recovery (Brandt & Stolle, 2021; Quandt & McCabe, 2017). In fragile environments, where formal forest governance may be weakened, trees on farms can represent an important component of ecological and socioeconomic resilience by providing locally accessible sources of food, fuelwood, income, and environmental services. Consequently, monitoring trees outside forests should be considered an important component of post-conflict environmental assessment and recovery planning.

Overall, the results show that tree cover on agricultural land in the study area increased over the period 2016–2024, primarily through conversions from cropland and, to a lesser extent, barren land. Several contextual mechanisms—including development interventions, farmer decision-making, land abandonment, and conflict-related land use dynamics—could plausibly have contributed to these patterns, but their relative roles cannot be determined from the present remote sensing analysis. Future research integrating field observations, household surveys, and higher-resolution imagery would help clarify the relative contributions of deliberate tree planting versus passive regeneration and assess the long-term sustainability of the observed trends.

These findings have important practical implications for land management and rural development in fragile contexts. The observed expansion of tree cover within agricultural land highlights the increasing presence of woody vegetation in these landscapes. Previous studies have shown that agroforestry systems can enhance soil fertility, regulate microclimates, and provide food, fuelwood, and income (Cyamweshi et al., 2023; Fuchs et al., 2022; Quandt & McCabe, 2017). In eastern Afghanistan, where pressure on remaining natural forests has historically been high (Ingalls & Mansfield, 2017; Reddy & Saranya, 2017), increasing tree cover on farms could potentially reduce reliance on natural forest resources by providing tree products locally, although this relationship was not directly assessed in the present study. These findings therefore highlight the potential value of supporting smallholder-based tree systems as part of broader land management and climate adaptation strategies. Such support could include secure land tenure, improved market access, and locally adapted extension services, although the effectiveness of these measures would require further socioeconomic investigation.

## Study limitations

Despite the strong classification performance and consistent patterns observed across the study period, several limitations should be acknowledged. First, field-based ground-truth data were not available. Due to security constraints and restricted access to parts of eastern Afghanistan, field surveys and ground validation were not feasible. Validation therefore relied on visual interpretation of high-resolution historical imagery available in Google Earth Pro. Although this provided a practical reference source in a conflict-affected environment, visual interpretation may not fully resolve spectrally and structurally similar land cover classes, particularly dense cropland, orchards, and natural woody regeneration. Consequently, some uncertainty in class discrimination remains.

Second, the 3 m spatial resolution and available spectral information of PlanetScope imagery enabled tree cover to be mapped at relatively fine spatial scales but did not permit reliable discrimination between tree species or between deliberately established trees and naturally regenerated woody vegetation. Future studies could improve class discrimination by incorporating multi-season imagery and phenological information, object-based texture metrics, imagery with additional spectral bands, and field-based reference observations where feasible.

Third, the study focused on land cover transitions and did not incorporate household-level socioeconomic information, such as land tenure, farm management practices, access to irrigation, market conditions, or farmers’ motivations for tree planting. The underlying socioeconomic and institutional drivers of tree cover change therefore could not be directly determined. Household surveys and field observations would be required to evaluate the potential mechanisms proposed in the ‘Discussion’ section.

Finally, the study covered the period from 2016 to 2024 and was conducted within delineated agricultural areas of four districts in Nangarhar Province rather than across the entire province or the whole of Afghanistan. The results therefore describe medium-term changes within the analysed AOIs and should not be generalised to the country as a whole. Continued monitoring beyond 2024 would help determine whether the observed increases in tree cover represent a persistent trajectory or shorter-term fluctuations.

## Conclusion

This study assessed changes in tree cover on agricultural land across four districts in eastern Afghanistan between 2016 and 2024 using high-resolution satellite imagery and GIS-based analysis. The results demonstrate a clear overall increase in tree cover within the analysed agricultural areas, from 369.26 ha in 2016 to 502.58 ha in 2024, representing a net gain of approximately 133 ha. Land use transition analysis showed that this increase occurred primarily through conversions from cropland to tree cover and, to a lesser extent, from barren land, while transitions involving built-up areas were limited. The observed expansion may reflect deliberate tree establishment, natural woody regeneration, or a combination of both; however, their relative contributions cannot be determined from the available remote sensing data. Overall, the findings demonstrate that substantial changes in tree cover can occur within agricultural landscapes even in a wider context of natural forest degradation. They also demonstrate the value of high-resolution remote sensing and post-classification change detection for monitoring trees outside forests in conflict-affected and data-scarce regions.

## Supplementary information

Below is the link to the electronic supplementary material.

ESM 1 (DOCX 3.02 MB)

## Data Availability

The datasets generated and analysed during the current study are available from the corresponding author upon reasonable request. PlanetScope imagery was obtained through Planet Explorer under an academic licence and is subject to Planet's data access policies. Google Earth Pro historical imagery was used as reference data for validation.
