# Supplementary material for: Tree cover increases on agricultural land amid continued landscape-level deforestation: a case study from eastern Afghanistan
Source: Environ Monit Assess. 2026 Oct 16;198(11):1203. doi: 10.1007/s10661-026-16018-1 (PMC13672257; doi:10.1007/s10661-026-16018-1)
Supplement: Supplementary file 1 [file 10661_2026_16018_MOESM1_ESM.docx]

**Supplementary information**

**Fig. S1** Sankey diagrams illustrating post-classification land-cover transitions between 2016–2020 across the four study districts. Flow widths are proportional to the area of land-cover conversion

**Fig. S2** Sankey diagrams illustrating post-classification land-cover transitions between 2020–2024 across the four study districts. Flow widths are proportional to the area of land-cover conversion

Table S1. PlanetScope satellite image acquisition dates and image IDs for the four study regions.

| **Region** | **Year** | **Acquisition Date** | **Image IDs** |
| --- | --- | --- | --- |
| Dehbala | 2016 | 2016-08-15 | 20160815_051041_0e0d, 20160815_051042_0e0d, 20160815_051043_0e0d, 20160815_051132_0e0f, 20160815_051133_0e0f, 20160815_051146_0e16, 20160815_051147_0e16, 20160815_051148_0e16 |
| Dehbala | 2020 | 2020-08-13 | 20200813_033324_1048, 20200813_033325_1048, 20200813_033326_1048, 20200813_054106_103b, 20200813_054258_1014, 20200813_054259_1014, 20200813_054300_1014 |
| Dehbala | 2024 | 2024-08-28 | 20240828_052503_73_24b4, 20240828_052505_91_24b4, 20240828_061757_55_2511, 20240828_061759_69_2511, 20240828_061801_84_2511, 20240828_062707_74_2498, 20240828_062709_55_2498 |
| Dara Noor | 2016 | 2016-08-15 | 20160815_051033_0e0d, 20160815_051034_0e0d |
| Dara Noor | 2020 | 2020-08-05 | 20200805_052728_0e3a, 20200805_054848_80_1065, 20200805_054850_33_1065 |
| Dara Noor | 2024 | 2024-08-18 | 20240818_052417_03_24c4 |
| Nazyan | 2016 | 2016-08-23 | 20160823_044647_0c76, 20160823_044648_0c76 |
| Nazyan | 2020 | 2020-08-14 | 20200814_061141_61_1064, 20200814_061143_62_1064 |
| Nazyan | 2024 | 2024-08-18 | 20240818_053916_16_2465, 20240818_053918_01_2465 |
| Pachir Aw Agam | 2016 | 2016-09-06 | 20160906_051035_0e19, 20160906_051036_0e19, 20160906_051037_0e19, 20160906_051332_0e26, 20160906_051333_0e26, 20160906_051334_0e26 |
| Pachir Aw Agam | 2020 | 2020-08-15 | 20200815_033438_0f4d, 20200815_033439_0f4d, 20200815_033440_0f4d, 20200815_051755_24_2277, 20200815_051757_46_2277, 20200815_051759_67_2277 |
| Pachir Aw Agam | 2024 | 2024-08-23 | 20240823_052408_83_24ca, 20240823_052411_03_24ca |

Table S2. SVM hyperparameters used for the district-year land-cover classifications.

| District | Year | SVM type | Kernel | C | Gamma (γ) | Maximum samples per class |
| --- | --- | --- | --- | --- | --- | --- |
| Dara Noor | 2016 | C-SVC | RBF | 32768 | 0.12 | 500 |
| Dara Noor | 2020 | C-SVC | RBF | 128 | 2.00 | 500 |
| Dara Noor | 2024 | C-SVC | RBF | 181.01 | 0.50 | 500 |
| Dehbala | 2016 | C-SVC | RBF | 8192 | 0.35 | 500 |
| Dehbala | 2020 | C-SVC | RBF | 23170.47 | 0.03 | 500 |
| Dehbala | 2024 | C-SVC | RBF | 512 | 2.00 | 500 |
| Nazyan | 2016 | C-SVC | RBF | 2048 | 0.03 | 500 |
| Nazyan | 2020 | C-SVC | RBF | 512 | 0.71 | 500 |
| Nazyan | 2024 | C-SVC | RBF | 45.25 | 1.00 | 500 |
| Pachir Aw Agam | 2016 | C-SVC | RBF | 11585.24 | 0.02 | 500 |
| Pachir Aw Agam | 2020 | C-SVC | RBF | 32768 | 0.04 | 500 |
| Pachir Aw Agam | 2024 | C-SVC | RBF | 256 | 1.41 | 500 |

Table S3. Land cover areas in 2016 (ha)

|  | Tree | Cropland | Developed Area | Water | Barren Land |
| --- | --- | --- | --- | --- | --- |
| Dehbala | 216.96 | 1348.28 | 263.54 | 247.32 | 49.01 |
| Nazyan | 12.82 | 210.18 | 103.37 | 40.26 | 46.48 |
| Pachir Aw Agam | 126.73 | 1345.45 | 247.60 | 166.03 | 5.06 |
| Dara Noor | 12.75 | 192.11 | 43.62 | 7.18 | 20.64 |
| Total | 369.26 | 3096.02 | 658.13 | 460.79 | 121.19 |

Table S4. Land cover areas in 2020 (ha)

|  | Tree | Cropland | Developed Area | Water | Barren Land |
| --- | --- | --- | --- | --- | --- |
| Dehbala | 184.93 | 1519.70 | 154.42 | 204.22 | 61.84 |
| Nazyan | 34.36 | 209.58 | 83.00 | 61.52 | 24.65 |
| Pachir Aw Agam | 190.65 | 1248.62 | 254.41 | 169.70 | 27.49 |
| Dara Noor | 13.12 | 205.16 | 37.39 | 6.88 | 13.75 |
| Total | 423.06 | 3183.06 | 529.22 | 442.32 | 127.73 |

Table S5. Land cover areas in 2024 (ha)

|  | Tree | Cropland | Developed Area | Water | Barren Land |
| --- | --- | --- | --- | --- | --- |
| Dehbala | 226.88 | 1370.21 | 229.72 | 231.16 | 67.14 |
| Nazyan | 70.34 | 135.35 | 91.77 | 87.96 | 27.69 |
| Pachir Aw Agam | 183.80 | 1287.90 | 199.78 | 191.82 | 27.57 |
| Dara Noor | 21.56 | 203.13 | 38.02 | 9.21 | 4.38 |
| Total | 502.58 | 2996.59 | 559.29 | 520.15 | 126.78 |

Table S6. Percentage of land-cover classes in each district for 2016, 2020, and 2024

| District | Year | Tree | Cropland | Developed Area | Water | Barren |
| --- | --- | --- | --- | --- | --- | --- |
| Dara Noor | 2016 | 4.61 | 69.53 | 15.79 | 2.60 | 7.47 |
| Dara Noor | 2020 | 4.75 | 74.25 | 13.53 | 2.49 | 4.98 |
| Dara Noor | 2024 | 7.80 | 73.52 | 13.76 | 3.33 | 1.59 |
| Dehbala | 2016 | 10.21 | 63.45 | 12.40 | 11.64 | 2.31 |
| Dehbala | 2020 | 8.70 | 71.51 | 7.27 | 9.61 | 2.91 |
| Dehbala | 2024 | 10.68 | 64.48 | 10.80 | 10.88 | 3.16 |
| Pachir Aw Agam | 2016 | 6.70 | 71.16 | 13.09 | 8.78 | 0.27 |
| Pachir Aw Agam | 2020 | 10.08 | 66.03 | 13.45 | 8.97 | 1.45 |
| Pachir Aw Agam | 2024 | 9.72 | 68.11 | 10.57 | 10.14 | 1.46 |
| Nazyan | 2016 | 3.10 | 50.88 | 25.02 | 9.75 | 11.25 |
| Nazyan | 2020 | 8.32 | 50.73 | 20.09 | 14.89 | 5.97 |
| Nazyan | 2024 | 17.03 | 32.76 | 22.21 | 21.29 | 6.70 |
